# Retrosplenial Cortical Contributions to Anterograde and Retrograde Memory in the Monkey

**DOI:** 10.1093/cercor/bhw054

**Published:** 2016-03-05

**Authors:** Mark J. Buckley, Anna S. Mitchell

**Affiliations:** Department of Experimental Psychology, Oxford University, OxfordOX1 3UD, UK

**Keywords:** amnesia, cingulate cortex, learning, lesion, macaque, retention

## Abstract

Primate retrosplenial cortex (RSC) is important for memory but patient neuropathologies are diffuse so its key contributions to memory remain elusive. This study provides the first causal evidence that RSC in macaque monkeys is crucial for postoperative retention of preoperatively and postoperatively acquired memories. Preoperatively, monkeys learned 300 object-in-place scene discriminations across sessions. After RSC removal, one-trial postoperative retention tests revealed significant retrograde memory loss for these 300 discriminations relative to unoperated control monkeys. Less robust evidence was found for a deficit in anterograde memory (new postoperative learning) after RSC lesions as new learning to criterion measures failed to reveal any significant learning impairment. However, after achieving ≥90% learning criterion for the postoperatively presented novel 100 object-in-place scene discriminations, short-term retention (i.e., measured after 24 h delay) of this well-learnt set was impaired in the RSC monkeys relative to controls. A further experiment assessed rapid “within” session acquisition of novel object-in-place scene discriminations, again confirming that new learning per se was unimpaired by bilateral RSC removal. Primate RSC contributes critically to memory by supporting normal retention of information, even when this information does not involve an autobiographical component.

## Introduction

Remembering personal events and episodes helps us to define our sense of self. Retrosplenial cortex (RSC) is suitably positioned to be involved in remembering these autobiographical memories given its anatomical links with both the extended hippocampal-diencephalic system and with the frontal lobes (Vogt et al. 1987; Kobayashi and Amaral 2003, 2007; Vann et al. 2009; Aggleton 2012). Amnesic patients exhibit significant glucose hypometabolism in the posterior cingulate region, centered in the RSC, from the early stages of Alzheimer's disease, as do the patient population with amnestic Mild Cognitive Impairment (Minoshima et al. 1997; Nestor et al. 2003). Patients with strokes or tumors affecting RSC can also have retrograde and anterograde amnesia and present difficulties with spatial navigation and orientation (Valenstein et al. 1987; Rudge and Warrington 1991; Takahashi et al. 1997; Maguire 2001). However, in all of these studies with amnesic patients the damage is never limited to RSC. Imaging studies using healthy volunteers have also been unable to illuminate the precise function of RSC because of a near ubiquitous activation in RSC across a broad range of disparate spatial and episodic memory tasks. However, clues do arise from meta-analyses of neuroimaging studies that, for example, have indicated higher RSC activation when subjects retrieve autobiographical memories (Maddock 1999; Spreng et al. 2009) and process landmark information (Maguire 2001; Spiers and Maguire 2006; Auger et al. 2012; Mullally et al. 2012; Auger and Maguire 2013). Therefore, in order to understand the crucial contributions of the RSC to memory and cognition, the study of selective surgical lesions using in vivo animal models are essential.

Animal rodent models have confirmed that lesions to the RSC impair spatial memory (Vann and Aggleton 2002; Vann et al. 2003; Harker and Whishaw 2004; Haijima and Ichitani 2008). However, the extent of damage within RSC itself and the extent of unintended damage to the cingulum bundle has long been a recognized issue of contention underlying variable results (Aggleton and Vann 2004; Harker and Whishaw 2004). In contrast, in macaques there have been no previous lesions studies of RSC.

Given the aforementioned proposed involvement of the RSC in both retrieval of autobiographical memories and in processing of spatial (e.g., landmark) information, we adopted an object-in-place learning task that incorporates elements of both spatial and episodic memory without being explicitly autobiographical in nature (Gaffan 1994; Mitchell and Gaffan 2008; Mitchell et al. 2008; Murray and Wise 2010). Further, this task allowed us to assess the impact of RSC lesions on both retention of preoperatively acquired memories (retrograde memory) and on new learning (anterograde memory). We assessed mnemonic ability both before and after RSC lesions thereby allowing for a more sensitive “within-subject” comparison of preoperative versus postoperative performance to be derived that is typically not possible in human neuropsychological investigations. We were also able to equate preoperative exposure to the object-in-place scene discrimination stimuli across subjects, which is also impossible in clinical studies. Importantly, our critical measure of retrograde memory was a one-trial per problem postoperative retention test which provides a measure of retention of preoperatively acquired memories uncontaminated by postoperative relearning confounds (Dean and Weiskrantz 1974; Gaffan 1993a; Buckley et al. 2004, 2008; Mitchell and Gaffan 2008; Mitchell et al. 2008).

## Materials and Methods

### Animals

Fourteen naïve rhesus monkeys (*Macaca mulatta*, 13 male, aged between 3.2 and 5 year at the beginning of behavioral training) participated. Thirteen monkeys participated in Experiments 1, 2, and 3; 4 male monkeys (RSC1–RSC4) formed the group with selective lesions to RSC, and 9 monkeys (CON1–CON9) were unoperated controls. Data from 5 of the unoperated control monkeys (CON1–CON5) had been previously published (Mitchell et al. 2008). All 13 monkeys learned the same 300 object-in-place scene discrimination problems divided into the same 3 preoperatively acquired sets of 100 problems each; they all performed the same preoperative and postoperative one-trial retention tests and relearning; and they all learned the same 100 novel object-in-place scene discriminations in postoperative acquisition spread across sessions. The remaining male monkey, RSC5, participated in Experiment 4 only, which involved a within-subject preoperative verses postoperative (after bilateral RSC ablations) assessment of rapid, within-session learning of novel object-in-place scene discriminations. All experimental procedures were performed in compliance with the United Kingdom Animals (Scientific Procedures) Act of 1986. A Home Office (UK) Project License, obtained after review by the University of Oxford Animal Care and Ethical Review Committee, licensed all procedures. The monkeys were socially housed together in same sex groups of between 2 and 6 animals. The housing and husbandry were in compliance with the guidelines of the European Directive (2010/63/EU) for the care and use of laboratory animals.

### Apparatus

The computer-controlled test apparatus was identical to that previously described (Mitchell et al. 2007). Briefly, monkeys sat in a transport box fixed to the front of a large touch-sensitive color monitor that displayed the visual stimuli for all of the experiments. Monkeys reached out through the bars of the transport box to respond on the touchscreen and collect their food reward pellets from a hopper that were automatically dispensed by the computer. Monkeys were monitored remotely via closed circuit cameras and display monitors throughout the testing period.

### Stimuli

**Figure 1.**
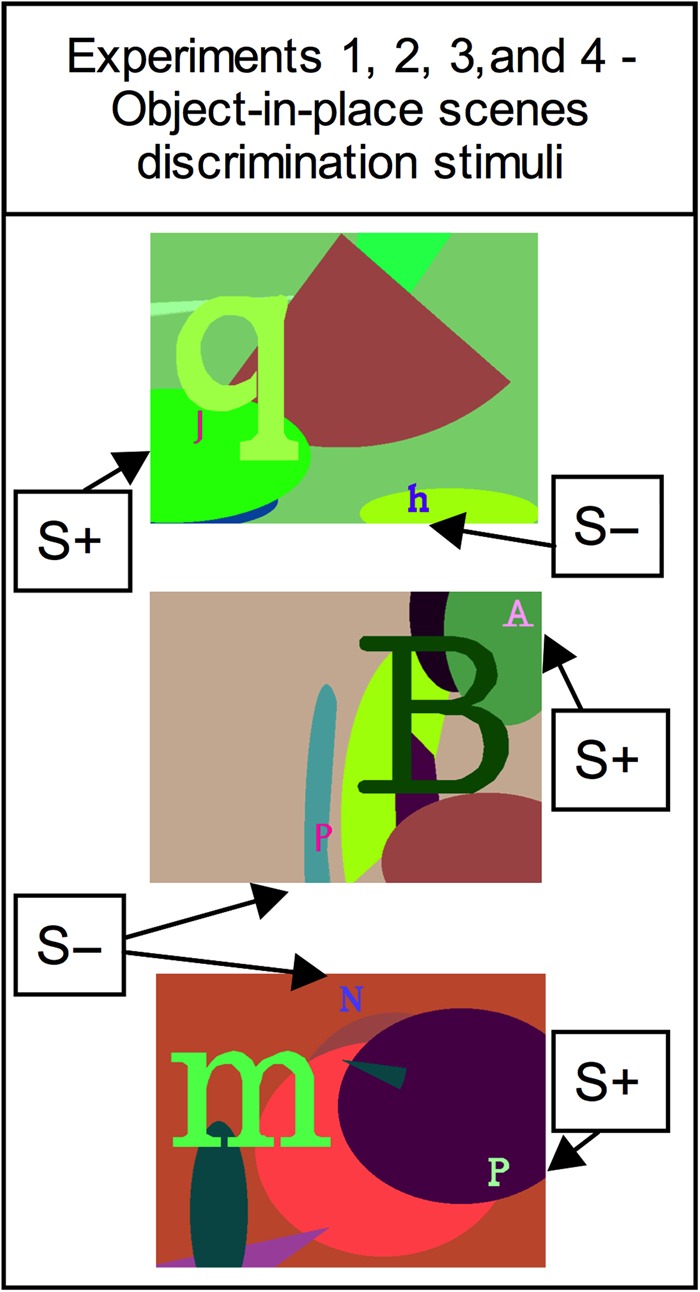
Three examples of object-in-place scene discrimination stimuli used in the 4 experiments in this study. The monkey responds to each “scene” by touching one of the 2 typographic foreground objects. One of the 2 foreground objects in each “scene” denoted by “S+” is arbitrarily designated as correct (reward). The “S−” indicates the locations of the unrewarded foreground objects in each “scene”. The locations and identities of the foreground objects are fixed within each scene, but vary across scenes.

The stimuli used in the object-in-place scene discrimination task were identical to those used by Gaffan (1994). Briefly, the stimulus for each trial consisted of an artificially constructed “scene” (see Fig. 1 for 3 examples). Each scene comprised a complex multi-colored background upon which 2 foreground alphanumeric characters were superimposed. The scenes were unique because they varied in several attributes determined at random when the scene was generated, namely: (1) the background color of the screen; (2) the color, size, orientation, and location of ellipse components of the background; (3) the identity, color, size, and location of one large typographic character that also formed part of the background and which was clearly distinct in size from the 2 small foreground objects; and (4) the identity, color, and location of 2 foreground alphanumeric “objects” unique to that scene. All the colors of the aforementioned components were randomly assigned with the constraint that the foreground objects should be visible (that is, there was a minimum separation in color space between the colors of a foreground object and the color of any element of its local background). From the animal's perspective (after task familiarization) each scene had only 2 viable alternatives to choose within it, namely the 2 foreground objects, for which one was arbitrarily assigned as correct (rewarded) for that scene and the other incorrect (nonrewarded). The location and identity and reward-status of the 2 foreground objects were different between scenes but unchanging within any given scene. Hence the scene provided a context, helpful for the animal to learn which foreground object was correct within each particular scene.

#### Experiment 1: Retrograde Memory: Assessment of Postoperative Retention of Preoperatively Acquired Object-in-Place Scene Discriminations

##### Behavioral pretraining

Pretraining followed a previously published protocol using the stimuli described above (Mitchell et al. 2007a). The behavioral training began once the monkeys were reliably touching the foreground objects when presented with a new discrimination scene and completing 50 trials a day with minimal accuracy errors (i.e., touching any location on the screen other than the foreground alphanumeric characters).

##### Behavioral training on 3 preoperatively acquired sets of 100 problems (Sets A, B, and C)

Once pretraining had completed, initial behavioral training involved all monkeys learning their first 100 object-in-place scene discriminations with the number of discrimination problems given in each session gradually increasing to 100, based on each monkey's performance. Once all monkeys were reliably completing daily sessions involving learning these first 100 object-in-place discriminations then preoperative training in the first set (i.e., Set A) commenced. Preoperative training of the first 100 object-in-place scene discrimination problems (i.e., Set A) involved the monkeys being exposed to this first set of all 100 problems from their first training session. A touch to the correct object in the scene for the current trial caused the object to flash for 2 s within the scene while the incorrect object stayed on screen along with the colorful visual background scene, then the screen blanked and a reward pellet was delivered into the hopper. A touch to the incorrect object in the scene for the current trial caused the screen to blank immediately, no reward was given and a delay of 10 s followed before the start of the correction procedure for the given trial. In the event of an incorrect choice (i.e., to the wrong object) in any scene a correction procedure for that particular object-in-place discrimination was then administered. The correction procedure involved the same visual scene being re-presented with only the correct object present within the scene to produce a forced-choice scenario wherein only one response, a correct (rewarded) one, was now possible.

Touches anywhere else in the scene caused the screen to blank and the same correction procedure was repeated. When the monkey completed the final correct trial of a session, the lunch box opened automatically, and the monkey received the large food reward, consisting of primate chow, and a variety of fruits and nuts, and was allowed time to eat the food before being returned to the home enclosure. Therefore the monkeys learnt, across several days, all 100 problems in Set A with each of the 100 object-in-place problems in Set A presented day after day until the whole set was learned to criterion. Hence, this learning paradigm incorporated a delay between consecutive training sessions of at least 24 h so the task relied on both within-session and between-session learning. After Set A was learnt to criterion the procedure was repeated for a new set of 100 novel object-in-place problems (Set B) and after Set B was learnt to criterion the procedure was repeated for another new set of 100 novel object-in-place problems (Set C). All monkeys saw the same discrimination problems. They learnt each set of 100 discriminations until they attained a performance criterion of ≥90% correct in each of 2 consecutive sessions (except CON8 who during learning of Set A attained >90% correct averaged across 2 consecutive sessions). Consequently, Set A was learnt at least 3–4 months prior to neurosurgery, Set B was learnt 2—3 months prior to neurosurgery and Set C was learnt within the month prior to neurosurgery.

##### One-trial preoperative and postoperative retention tests

Our key aim in Experiment 1 was to test retrograde memory for preoperatively learnt object-in-place scene discriminations (i.e., postoperative retention of the 3 sets of preoperatively learnt problems). For analyses of retention, in order to avoid confounding postoperative tests of retention with postoperative tests of new learning one needs to restrict retention analyses to one-trial postoperative retention tests (i.e., each problem tested only once postoperatively). In order to compare one-trial postoperative retention tests with an equivalent measure in each animal, prior to neurosurgery we assessed their retention performance using a one-trial preoperative retention test of all 300 problems.

After reaching the behavioral performance criterion for preoperative learning on the third set (Set C), the set of 300 one-trial preoperative retention tests (i.e., one trial per problem) was conducted in successive days. The first of these days consisted of “familiarization”; the monkeys saw 100 novel object-in-place scene discrimination trials using the task, but with only one random alphanumeric character presented against the colorful “scene” background (these trials being identical to correction procedure trials). The monkeys had to touch the single alphanumeric character to receive a reward. Responses to anywhere else on the screen – “reaching-accuracy errors” immediately ended the presentation, and then the same trial was re-presented after a 10 s delay. This familiarization procedure (also used in the same way postoperatively) allowed us to confirm, prior to commencing the retention test proper, that the monkeys′ motivation for testing had not altered (e.g., including after the extended break from testing imposed as a consequence of postoperative recovery). On the second day, the monkeys were presented with Set A using the same testing methods experienced during preoperative training. On the third day, Set B was presented, and on the fourth day, Set C was presented. These 3 final consecutive days of testing (days 2–4) constituted the one-trial preoperative retention test. Four monkeys then received bilateral RSC lesions (Group RSC; RSC1–RSC4) and these were compared with 9 unoperated controls (Group CON). Both groups rested for the same duration of time (at least 12 days) prior to resuming testing at which point an identical one-trial postoperative retention test per problem (preceded by the familiarization session) spread over 4 consecutive days was given for all 300 object-in-place scene discriminations.

#### Experiment 2: Postoperative Relearning of the 300 Preoperatively Acquired Object-in-Place Scene Discriminations

After the postoperative retention test, each monkey continued testing for 3 further repeats (cycles) of the one-trial postoperative retention test described above, using Sets A, B, C (except CON8 who had only one further cycle of the one-trial retention test). This testing period did not include any further sessions using familiarization. This testing was designed to assess postoperative relearning of the preoperatively acquired material. This experiment was therefore neither a pure assessment of retrograde nor anterograde memory per se as the learning may be considered relearning or new learning.

#### Experiment 3: Anterograde Memory Assessment: Postoperative Learning and Retention of a new set of 100 Novel Object-in-place Scene Discriminations not Seen Preoperatively

We then assessed new postoperative acquisition of a set of 100 novel object-in-place scene discriminations (Set D) that the monkeys had not been exposed to previously at any stage. The type of stimuli, rewards, and procedure used in this postoperative learning task were identical to those previously described for preoperative learning of Sets A, B, C. This learning of novel object-in-place scene discriminations “across” sessions was done in order to assess the effect of RSC lesions on anterograde memory loss. As with learning of Sets A, B, C, this learning paradigm also incorporated a delay between consecutive training sessions of at least 24 h and therefore involved both within-session and between-session learning. New learning ability was measured as the number of errors each group made to learn 100 novel discriminations (postoperative exposure only) to ≥90% correct across 2 consecutive days. As our hypotheses concerned the potential presence of differential effects of RSC lesions on retrograde versus anterograde amnesia we probed new learning in greater detail and assessed the intermediate numbers of errors each group made to learn these 100 novel discriminations to ≥65% correct, ≥70% correct, ≥75% correct, ≥80% correct, and ≥85% correct criteria (in addition to the ≥90% criteria mentioned above which is the standard assessment measure on our task). Furthermore, we also assessed one-trial postoperative retention of this one set of postoperatively acquired 100 novel object-in-place discriminations in Set D by comparing the number of errors made by each monkey in the session on the day after (i.e., 24 h delay) they attained ≥90% correct for the first time. Further, these analyses did not include any analyses of any correction procedure errors, as correction procedures were also not used in our retention tests of Sets A, B, C.

#### Experiment 4: Anterograde Memory Assessment: Rapid Within-Session Postoperative New Learning of Sets of 10 New Object-in-Place Scene Discriminations Each Day

This experiment assessed more rapid postoperative acquisition of new smaller sets of object-in-place scene discriminations that were learnt solely within each daily testing session (because a new set of discriminations was given every day), prior to and after a bilateral RSC lesion. For this experiment, one new monkey (RSC5) participated in this experiment exclusively and learning ability was compared between its preoperative versus postoperative performance scores. The testing apparatus was identical to that described above. The object-in-place scene discriminations were similar to the 3 examples in Figure 1.

The monkey was required to learn a novel set of 10 new object-in-place scene discriminations within each testing session, by being exposed to an initial run through the set of 10 novel discriminations followed by 7 repetitions of this set of 10 discriminations within the session (in the same order each time). On the next daily testing session, a novel set of 10 discriminations was presented for within-session learning in the same fashion as above, and so on. During daily learning, performance on the first presentation of the 10 novel discriminations (i.e., run 1) is accordingly at chance, as the monkey has no information about which is the correct object to choose on the very first exposure to each discrimination. Then through subsequent repetitions of the same discriminations within the session (i.e., runs 2–8) the monkey subsequently learns the discriminations rapidly. Once stable learning is established within each session across several weeks of testing with a novel set of 10 discrimination problems presented in each testing session, a monkey has a rest period of 2 weeks (equivalent in duration to a “postoperative rest”) then a preoperative performance test for 13 days is conducted. For days 1 and 2 of the performance test, the monkey receives one session of 5 novel object-in-place problems (with 8 repetition runs within the session), again with novel problems used each day. Then, for days 3–13 the monkey receives their preoperative performance test with 10 novel problems each day and 8 repetition runs within each session. The preoperative performance test data is analyzed from days 4–13. After surgery and 2 weeks postoperative rest, an identical method was followed to obtain postoperative within-session learning data averaged over the last 10 performance sessions (days 4–13). Proficiency in preoperative and postoperative within-session learning in this task is expressed as average percent errors in repetition runs 2–8 across the final 10 sessions of testing (i.e., days 4–13).

##### Surgery

Five monkeys in the current experiments received neurosurgery involving bilateral ablations to the RSC (RSC1–RSC5). Neurosurgical procedures were performed in a dedicated operating theatre under aseptic conditions and aided by an operating microscope. Steroids (methylprednisolone, 20 mg/kg) were given the night before surgery intramuscularly (i.m.), and 4 doses were given 4–6 h apart (intravenously [i.v.] or i.m.) on the day of surgery to protect against intraoperative edema and postoperative inflammation. Each monkey was sedated on the morning of surgery with both ketamine (10 mg/kg) and xylazine (0.25–0.5 mg/kg, i.m.). Once sedated, the monkey was given atropine (0.05 mg/kg, i.m.) to reduce secretion, antibiotic (amoxicillin, 8.75 mg/kg) as prophylaxis against infection, opioid (buprenorphine 0.01 mg/kg, repeated twice at 4- to 6-h intervals on the day of surgery, i.v. or i.m.) and nonsteroidal anti-inflammatory (meloxicam, 0.2 mg/kg, i.v.) agents for analgesia, and an H2 receptor antagonist (ranitidine, 1 mg/kg, i.v.) to protect against gastric ulceration as a side effect of the combination of steroid and nonsteroidal anti-inflammatory treatment. The head was shaved and an intravenous cannula put in place for intraoperative delivery of fluids (warmed sterile saline drip, 5 mL/h/kg). The monkey was moved into the operating theatre, intubated, placed on sevoflurane anesthesia (1–4%, to effect, in 100% oxygen), and then mechanically ventilated. A hot air blower (Bair Hugger) allowed maintenance of normal body temperature during surgery. Heart rate, oxygen saturation of hemoglobin, mean arterial blood pressure, and tidal CO_2_, body temperature, and respiration rate were monitored continuously throughout the surgery.

##### RSC ablations

In each surgery, the monkey was placed in a stereotaxic head-holder and the head cleaned with alternating antimicrobial scrub and alcohol and draped to allow a midline incision. After opening the skin and underlying galea in layers, a large D-shaped bone flap was created in the cranium over the area of the operation and the dura over the posterior part of the hemisphere was cut and retracted to the midline. Veins draining into the sagittal sinus were cauterized and cut. The hemisphere was retracted with a brain spoon from the falx to enable access to the interhemispheric fissure. A small-gauge metal aspirator, insulated to the tip, was used to aspirate the tissue in the lower bank of the cingulate sulcus (PCC; Brodmann area 23), most ventral to the cingulate sulcus and into the RSC (Brodmann areas 29 and 30). Once the lesion to the exposed hemisphere was complete, the falx cerebri was cut and the contralateral hemisphere was ablated in a similar manner. When the lesions were complete, the dura was repositioned but not sewn, the bone flap was replaced and held with loose sutures, and the galea and skin were closed with sutures in layers. To reduce cerebral edema, mannitol (20%; a sugar alcohol solution; 1 mg/kg, i.v.) was administered slowly for 30 min while the monkey was still anesthetized. Then the monkey was removed from the head-holder and anesthesia discontinued. The monkey was extubated when a swallowing reflex was observed, placed in the recovery position in a separate cage within their homeroom, and monitored continuously. Normal posture was regained upon waking (waking times varied between 10 and 20 min after the discontinuation of the anesthesia); all monkeys were kept warm with blankets during this time. Operated monkeys rejoined their social groupings as soon as practical after surgery, during the second postoperative recovery day.

After all neurosurgery, each monkey was monitored continuously for at least 48 h. Postoperative medication continued in consultation with veterinary staff, including steroids (dexamethasone, 1 mg/kg, i.m.), once every 12 h for 3 days, then once every 24 h for 2 days; analgesia (buprenorphine, 0.01 mg/kg, i.m.) for 48 h; and antibiotic treatment (amoxicillin, 8.75 mg/kg, oral) for 5 days. Gastric ulcer protection (omeprazole, 5 mg/kg, oral and antepsin, 500 mg/kg, oral) commenced 2 days prior to surgery and continued postoperatively for the duration of other prescribed medications, up to 5 days.

##### Histology

After completion of all behavioral testing, each monkey with a lesion was sedated with ketamine (10 mg/kg), deeply anesthetized with intravenous barbiturate and transcardially perfused with 0.9% saline followed by 10% formalin. The brains were cryoprotected in formalin sucrose and then sectioned coronally on a freezing microtome at 50 µm thickness. A 1-in-10 series of sections was collected throughout the cerebrum; these were mounted on gelatin-coated glass microscope slides and stained with cresyl violet.

##### Assessment of the RSC lesions

**Figure 2.**
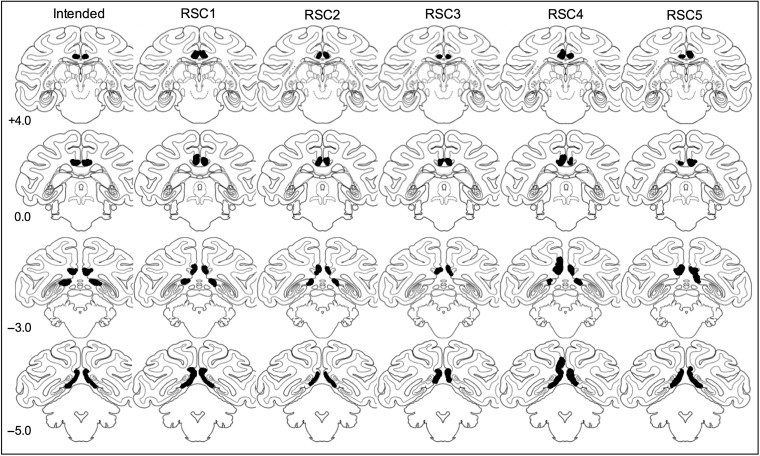
Schematic drawings of the intended bilateral retrosplenial cortex (RSC) ablations (first column) and ablations from the histology of the 5 monkeys (RSC1–5) with RSC damage (second–fifth columns) using the standard rhesus monkey brain (Saunders 2006). Numbers refer to the coronal sections from the atlas.

**Figure 3.**
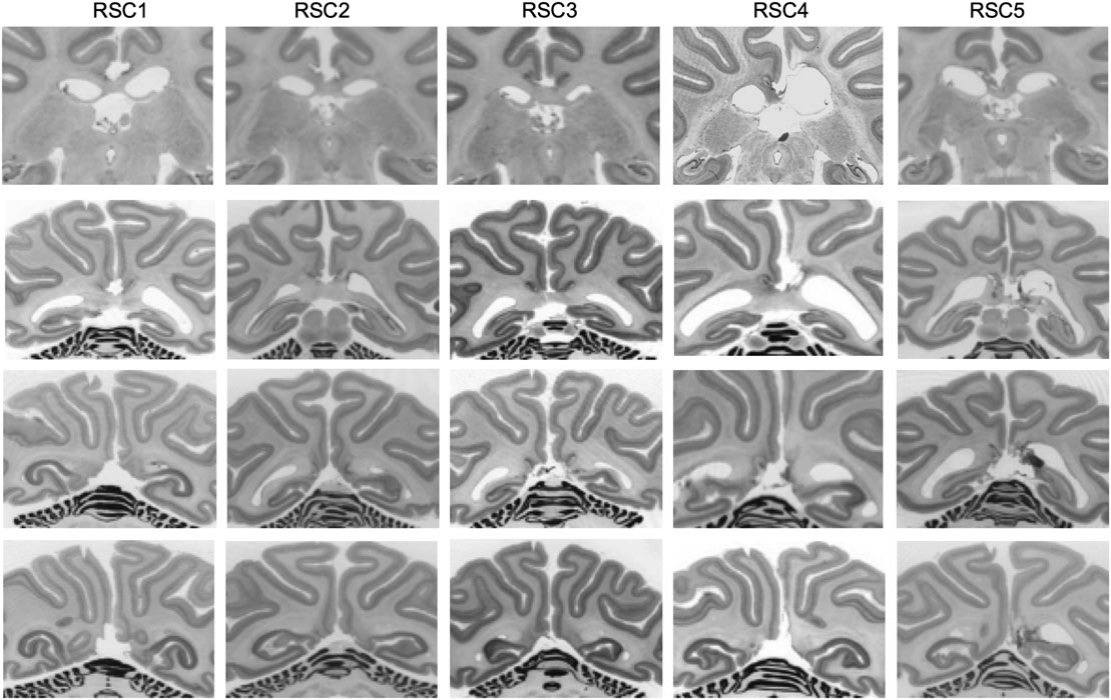
Photomicrographs from the histology of the 5 monkeys with RSC ablations. The coronal sections shown correspond as closely as possible to the rostrocaudal coordinates (line 1, A + 5.6; line 2, A + 2.6; line 3, A + 1.6; line 4, A + 0.6) from (Kobayashi and Amaral 2000). Four monkeys (RSC1, RSC2, RSC3, RSC4) participated in the combined preoperative and postoperative one-trial retention tests of 300 object-in-place scene discriminations, relearning and postoperative learning across sessions of 100 novel object-in-place scene discriminations (Experiments 1–3) and one monkey (RSC5) participated in postoperative learning within-sessions of new object-in-place scene discriminations (Experiment 4).

All monkeys received bilateral lesions that were intended to be limited to the RSC (Figs 2 and 3 for schematic drawings and photomicrographs of the lesions). The rostral limit of the intended lesion extended anteriorly within the posterior cingulate cortex approximately 15 mm from the end of the splenium of the corpus callosum; the lateral intended lesion limit extended to the white fibers of the cingulum bundle and the caudal ventral limit was the calcarine sulcus. In all cases, the intended RSC lesions also damaged parts of posterior cingulate cortex (area 23) running along the midline, and there was some sparing of lateral RSC in area 29 (located in the most lateral and caudal aspects of RSC above the splenium). One monkey, RSC4, sustained unilateral right hemisphere damage to the cingulum bundle and also had some damage to the splenium. One monkey, RSC3, sustained damage to the RSC above the splenuim only. All monkeys also had cell loss within the anterior thalamus, with the most noticeable changes to the anterodorsal and anteroventral subdivisions of the anterior thalamus complex as well as to the laterodorsal thalamic nucleus. This retrograde degeneration of cells in the anterior regions of the limbic thalamus is consistent with the dense neuroanatomical interconnections of these regions with the posterior cingulate and retrosplenial cortices in the rhesus macaque (Vogt et al. 1987; Aggleton et al. 2014).

## Results

### Experiment 1: Retrograde Memory: Assessment of Postoperative Retention of Preoperatively Acquired Object-in-Place Scene Discriminations

#### Reaching-Accuracy in Familiarization Stages Prior to Preoperative and Postoperative One-Trial Retention Tests (RSC Group vs. CON Group)

There was no difference between groups in the number of reaching-accuracy errors produced during the preoperative “familiarization” session conducted just prior to the preoperative performance test, [*F*_1,11_ = 2.95, *P* = 0.114] or during the preoperative retention test, [*F*_1,11_ = 1.12, *P* = 0.312]. The 2 groups did differ in the number of reaching-accuracy errors they produced during the postoperative “familiarization” session conducted just prior to performing the postoperative one-trial retention test, [*F*_1,11_ = 8.40, *P* = 0.015]. Monkeys with RSC ablations made more reaching-accuracy errors (M = 7.50, S.D. = 2.52) during this postoperative “familiarization” session than the unoperated controls (M = 3.11, S.D. = 2.52). However, the difference in number of reaching-accuracy errors between RSC and CON was not significant for the postoperative retention test itself, [*F*_1,11_ = 2.09, *P* = 0.176].

#### Assessment of Preoperative Learning and Retention Rates (RSC Group vs. CON Group)

The training and testing performance data for all monkeys in these experiments is presented in Table 1. Monkeys destined to receive bilateral lesions to RSC or to remain as unoperated controls (CON) did not differ in their preoperative learning ability. There were no differences in learning abilities of monkeys for the 3 sets (A, B, C) of 100 object-in-place scene discriminations across sessions, [*F*′s < 1.0]. The 2 groups also did not differ in their ability to remember the discriminations as measured by the total number of errors made in the preoperative one-trial retention test. One-way analysis of variance (ANOVA) of these errors made across the 2 groups confirmed there were no differences in preoperative retention errors for Set A, Set B, or for Set C, [*F*′s < 1.0]. In addition, the 2 groups did not differ in the number of days that intervened between the preoperative one-trial retention test and the postoperative one-trial retention test [group RSC, mean = 21, range = 17-26d; group CON, mean = 18, range = 15–24d. Furthermore, there were no differences between the 2 groups of controls (one-way ANOVA: initial learning of Set A, *F*_1,8_ = 2.36, *P* = 0.168; Set B, *F*_1,8_ = 1.03, *P* = 0.344, Set C, *F* < 1.0; Pre-op retention, Set A, *F*_1,8_ = 2.36, *P* = 0.168; Set B, *F* < 1.0, Set C, *F* < 1.0; or Post-op retention, Set A, *F* < 1.0; Set B, *F* < 1.0, Set C, *F*_1,8_ = 2.07, *P* = 0.193).

**Table 1:** Preoperative and postoperative performance of individual monkeys during learning and retention of sets of 100 object-in-place scene discriminations

	Preoperative training			Preoperative training			Preoperative retention test			Postoperative retention test			Postoperative new learning	
	Sessions			Errors			Errors			Errors			Sessions	Errors
Monkey	Set A	Set B	Set C	Set A	Set B	Set C	Set A	Set B	Set C	Set A	Set B	Set C	Set D	Set D
CON1	14	15	8	343	305	169	28	7	4	21	17	22	10	291
CON2	30	14	13	710	337	275	22	18	4	20	16	11	9	205
CON3	22	16	9	573	351	230	12	14	9	12	8	12	6	164
CON4	42	15	10	987	335	278	15	12	7	24	18	11	8	204
CON5	33	13	11	815	341	265	22	28	15	25	21	13	12	272
CON6	8	11	9	206	232	183	27	20	9	29	13	7	6	136
CON7	30	15	13	605	289	327	20	21	16	19	18	16	9	215
CON8	10	11	13	245	205	255	25	10	6	19	10	5	13	302
CON9	28	18	13	672	415	277	30	18	8	20	18	9	13	323
Mean	24.1	14.2	11	571.8	312.2	255.8	22.3	16.4	8.7	21	15.4	11.8	9.5	234.7
RSC1	27	17	24	731	380	410	23	13	6	24	22	13	13	289
RSC2	7	10	8	182	177	183	32	21	13	32	26	21	10	246
RSC3	15	12	8	367	288	138	18	7	11	16	19	8	8	191
RSC4	21	13	13	532	326	270	28	17	12	31	28	23	19	494
Mean	17.5	13	13	453	292.8	250.3	25.3	14.5	10.5	25.8	23.8	16.3	12.5	305

Note: The data shown are the total number of sessions required and errors made to learn Set A, Set B, and Set C to ≥90% correct across 2 consecutive sessions; the individual monkeys′ errors made in the preoperative one-trial retention test and the postoperative one-trial retention test for each of the 3 sets (A, B, C) of 100 object-in-place scene discriminations; sessions and errors to criterion of ≥90% correct across 2 consecutive sessions for new postoperative learning of set D, a novel set of 100 object-in-place scene discriminations, for unoperated control monkeys (CON1–CON9; CON1–CON5 are from a previously published study (Mitchell et al. 2008)) and monkeys with bilateral retrosplenial cortex ablations (RSC1–RSC4).

#### Preoperative Versus Postoperative One-Trial Retention Tests to Assess Retrograde Memory (RSC Group vs. CON Group)

**Figure 4.**
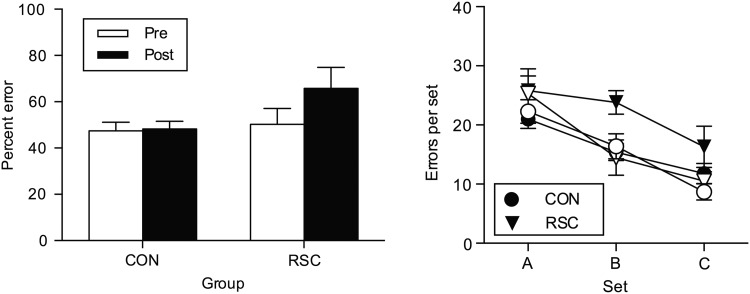
Preoperative and postoperative one-trial (per problem) retention test performance. Left, Total mean (+SEM) errors in memory retention for the unoperated controls (CON, *n* = 9) and bilateral retrosplenial cortex ablation (RSC, *n* = 4) monkeys during the preoperative (Pre, white bars) and postoperative (Post, black bars) one-trial retention tests summed over all 3 of the preoperatively learned sets (A–C). Right, The total mean (±SEM) errors per set made by unoperated control monkeys (CON, *n* = 9, circles; open circles = pre-op; black circles = post-op) and by bilateral retrosplenial cortex ablation monkeys (RSC, *n* = 4, triangles; open triangles = pre-op; black triangles = post-op) are shown for each of the 3 preoperatively learned sets (A–C) of 100 object-in-place scene discriminations each. Open symbols represent errors during the preoperative one-trial retention test; filled black symbols represent errors during the postoperative one-trial retention test.

To assess the overall effect of the RSC lesion on retrograde memory loss (i.e., postoperative testing on preoperatively learnt object-in-place problems), the number of errors produced in the one-trial postoperative retention tests (i.e., one trial only for each of the 100 object-in-place discrimination problems in each of the 3 sets [A, B, C]) was compared with the number of errors produced during the one-trial preoperative retention tests for each problem (Table 1). After surgery, the RSC group showed greater retrograde memory loss for the 300 problems compared with the CON group. A repeated-measures ANOVA with 2 within-subject factors “testing phase” (2 levels: Pre, Post) and “problem set” (3 levels: Set A, Set B, Set C), and one between-subject factor “Group” (2 levels: RSC, CON) revealed that significant main effects of testing phase, [*F*_1,11_ = 6.64, *P* = 0.026] and problem set, [*F*_2,22_ = 35.59, *P* < 0.001] were present, but the main effect of Group was not significant, [*F*_1,11_ = 2.30, *P* = 0.157]. However, the testing phase × Group interaction was found to be just significant, [*F*_1,11_ = 5.43, *P* = 0.040] (Fig. 4, left), signifying that the RSC group were impaired relative to the CON group in postoperative retention of preoperatively acquired problems. To explore whether the significant interaction may reflect a disproportionate deficit with respect to one or more problem sets, we inspected the “testing phase” × “Group” × “problem set” interaction; this was found not to be significant, [*F*_2,22_ = 2.16, *P* = 0.140]; the linear trend component of the 3-way interaction was also not significant, [*F* < 1], so we found no evidence for any temporal gradient of retrograde amnesia. Inspection of Figure 4 (right) shows that the greatest numerical difference between RSC and CON groups was in Set B; indeed the fact that the quadratic component of the 3-way interaction was significant, [*F*_1,11_ = 6.29, *P* = 0.029] reflects this. In summary, we only found statistical support for an overall deficit in retrograde amnesia in the RSC monkeys compared with CON monkeys without any significant statistical support from our dataset to indicate that the retrograde amnesia after the RSC damage in monkeys is temporally graded. Furthermore (see Figs 3 and 4), the controls made more “postoperative” errors than “preoperative” errors for Set C only, that is the set that was learnt closest in time to the 2-week “postoperative recovery” break despite being retested on all 3 preoperatively learned sets in the one-trial preoperative retention test that occurred just prior to their “postoperative recovery” break.

#### Meta-Analysis of Preoperative Versus Postoperative One-Trial Retention Tests to Assess Retrograde Memory (RSC Group vs. CON Group vs. ERh Group vs. MD+Fx Group)

**Figure 5.**
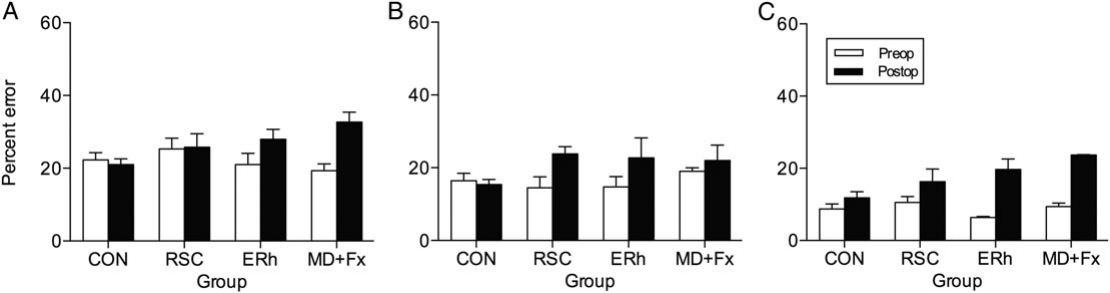
Preoperative and postoperative one-trial (per problem) retention test performance. (*A*) Total mean (+SEM) errors in memory retention during the preoperative (Pre, white bars) and postoperative (Post, black bars) one-trial retention tests for Set *A*; (*B*) for Set *B*; (*C*), for Set *C* for the unoperated controls monkeys (CON, *n* = 9), for bilateral retrosplenial cortex ablation monkeys (RSC, *n* = 4), for bilateral entorhinal cortex ablation monkeys (ERh, *n* = 3) and for bilateral neurotoxic lesions to magnocellular subdivision of the mediodorsal thalamus combined with bilateral fornix transection monkeys (MD + Fx, *n* = 3). The monkeys with ERh and MD + Fx lesions had been previously published (Mitchell et al. 2008).

We conducted a meta-analysis to examine if the retrograde memory performance of RSC-lesioned monkeys differed in degree from that of groups of monkeys with different lesions within an extended network of memory related brain structures. For this meta-analysis we compared the performance of the current RSC group with other groups of lesioned monkeys that had previously been assessed on the same measures, namely groups of monkeys with bilateral lesions to entorhinal cortex (Group ERh) or bilateral lesions to mediodorsal thalamus combined with fornix transection (Group MD + Fx) that have previously been published elsewhere (Mitchell et al. 2008). Comparative data across the 3 sets for each group are depicted in Figure 5.

For this meta-analysis, we first ran a repeated-measures ANOVA with 2 within-subject factors “testing phase” (2 levels: Pre, Post) and all 3 problem sets (3 levels: Set A, Set B, Set C), and one between-subject factor “Group” (4 levels: RSC group, CON group, ERh group, and MD + Fx group) which confirmed that significant main effects of testing phase, [*F*_1,15_ = 48.88, *P* < 0.001] and problem set, [*F*_2,30_ = 40.48, *P* < 0.001] were present, but no main effect of Group, [*F*_3,15_ = 2.07, *P* = 0.148]. There was no significant interaction of Group by problem set, [*F* > 1.0], although there was a significant interaction of testing phase × problem set, [*F*_3,30_ = 2.77, *P* < 0.035], indicating that between the 2 testing phases (pre-op vs. post-op), errors made across the 3 sets were significantly different.

Furthermore, with the inclusion of the 2 additional lesion groups into this meta-analysis, the testing phase × Group interaction became highly significant, [*F*_3,15_ = 8.90, *P* = 0.001] indicating significant differences between groups in their pre-lesion versus post-lesion performance. However, Bonferroni post hoc tests failed to show that any group was significantly different from the CON group in their overall mean preoperative versus postoperative retention scores (RSC vs. CON, “mean difference” = 10.17, *P* = 0.734; ERh vs. CON, mean difference = 8.33, *P* = 1.0; MD + Fx vs. CON, mean difference = 15.17, *P* = 0.263).

In order to explore the nature of changes in retention by monkeys with different lesions for the different sets, we ran further analyses on each set separately. A repeated-measures ANOVA for Set A (the first preoperatively learnt set) incorporating the within-subject factor “testing phase” (2 levels: Pre, Post) and the between-subject factor “Group” (4 levels: RSC group, CON group, ERh group, and MD + Fx group) revealed a significant testing phase × Group interaction, [*F*_3,15_ = 6.94, *P* = 0.004]. However, Bonferroni post hoc tests failed to show that any group was significantly different from the CON group in their mean preoperative versus postoperative retention scores (RSC vs CON, mean difference = 3.83, *P* = 1.0; ERh vs CON, mean difference = 2.83, *P* = 1.0; MD + Fx vs. CON, mean difference = 4.33, *P* = 1.0). The equivalent analysis on Set B also revealed a significant testing phase × Group interaction, [*F*_3,15_ = 4.23, *P* = 0.024]. However, Bonferroni post hoc tests also failed to show that any group were significantly different from the CON group in their mean preoperative versus postoperative retention scores (CON vs. RSC, mean difference = 3.18, *P* = 1.0; CON vs. ERh, mean difference = 2.72, *P* = 1.0; CON vs. MD + Fx, mean difference = 4.56, *P* = 1.0). Similarly, when applied to Set C the same analysis revealed a significant testing phase × Group interaction, [*F*_3,15_ = 4.44, *P* = 0.020]. However, Bonferroni post hoc tests again failed to show that any group were significantly different from the CON group in their mean preoperative versus postoperative retention scores (RSC vs. CON, mean difference = 3.15, *P* = 0.851; ERh vs. CON, mean difference = 2.78, *P* = 1.0; MD + Fx vs. CON, mean difference = 6.28 *P* = 0.084).

The meta-analysis taken together with the original analysis offers very little evidence to suggest that the RSC lesion effects differ markedly from that of the ERh or MD + Fx lesions. More work with more monkeys will be required to determine if the numerical trends (Fig. 5) are indicative of RSC lesions producing deficits in retrograde memory that are any more temporally selective than those which follow either from ERh or MD + Fx lesions.

### Experiment 2:Postoperative Relearning of the 300 Preoperatively Acquired Object-in-Place Discriminations (RSC Group vs. CON Group)

**Figure 6.**
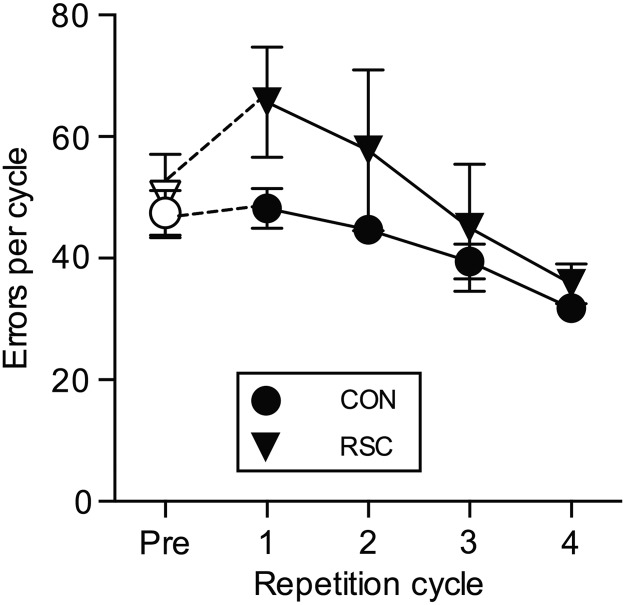
Postoperative relearning of preoperatively acquired problems. Total mean errors summed over all 3 of the preoperatively learned sets (A–C) for each group. Repetition cycles Pre and 1 are the preoperative and postoperative one-trail retention tests, respectively; Repetition cycles 2–4 are further repeats of the same postoperative one-trial retention test. Open symbols represent errors made during the preoperative one-trial retention test; filled black symbols represent errors made during the postoperative one-trial retention tests.

To assess the overall effect of the RSC lesion on relearning of information learnt prior to brain injury we next considered only the number of errors produced during 3 further repetitions (cycles) of the one-trial retention test (i.e., cycles 2–4) conducted immediately after the first cycle of 300 one-trial postoperative retention tests (Table 2). A repeated-measures ANOVA with 2 within-subject factors “problem set” (3 levels: Set A, Set B, Set C) and “repetition cycle” (3 levels: cycles 2–4) and the between-subject factor “Group” (2 levels: RSC group, CON group) showed a significant main effect of Repetition cycle, [*F*_2,20_ = 16.13, *P* < 0.001] reflecting relearning taking place in both groups (Fig. 6) and a significant main effect of problem set, [*F*_2,20_ = 10.41, *P* = 0.001]. There was no significant main effect of Group, [*F*_1,10_ = 1.22, *P* = 0.294] nor any significant interaction between either problem set × Group, [*F* < 1.0] or Repetition cycle × Group, [*F*_2,20_ = 1.32, *P* = 0.290] or problem set × repetition cycle × Group, [*F*_4,40_ = 2.00, *P* = 0.113]. This analysis considers relearning separate from the pure assessment of retention offered by the one-trial postoperative retention test (i.e., cycle 1). Even repeating the analysis above with cycle 1 included results in a nonsignificant main effect of Group, [*F*_1,10_ = 2.08, *P* = 0.180] and all of the aforementioned interactions remain nonsignificant. Therefore these analyses provide no evidence in support of the hypothesis that monkeys with bilateral RSC lesions are impaired at relearning of information learnt prior to brain injury. They are impaired at the initial postoperative test of retention of preoperatively learnt information but subsequent relearning of the same information is not impeded.

**Table 2:** Postoperative relearning

Monkey	Repetition cycle 2			Repetition cycle 3			Repetition cycle 4		
	Set A	Set B	Set C	Set A	Set B	Set C	Set A	Set B	Set C
CON1	17	9	15	12	10	19	11	9	13
CON2	12	13	10	14	10	7	13	10	10
CON3	15	10	8	17	7	6	10	4	5
CON4	15	16	13	24	14	10	18	6	7
CON5	19	14	18	21	16	8	14	15	11
CON6	21	23	9	17	11	9	20	11	5
CON7	15	14	21	20	14	19	11	10	13
CON8	28	12	6	–	–	–	–	–	–
CON9	19	14	16	11	9	11	9	9	10
Mean	17.9	13.9	12.9	17	11.4	11.1	13.3	9.3	9.3
RSC1	16	18	10	9	11	16	16	12	4
RSC2	37	30	28	30	19	26	14	11	20
RSC3	21	9	5	15	8	4	11	6	13
RSC4	27	13	17	21	10	11	10	12	14
Mean	25.3	17.5	15	18.8	12	14.3	12.8	10.3	12.8

Note: Postoperative relearning rates of each set during 3 further repetitions (repetition cycle 2–4) of the one-trial retention test immediately following the initial postoperative one-trial retention test.

### Experiment 3: Anterograde Memory Assessment: Postoperative Learning and Retention of a New Set of 100 Novel Object-in-Place Discriminations Not Seen Preoperatively (RSC Group vs. CON Group)

#### Postoperative New Learning-to-Criterion of 100 New Problems

**Figure 7.**
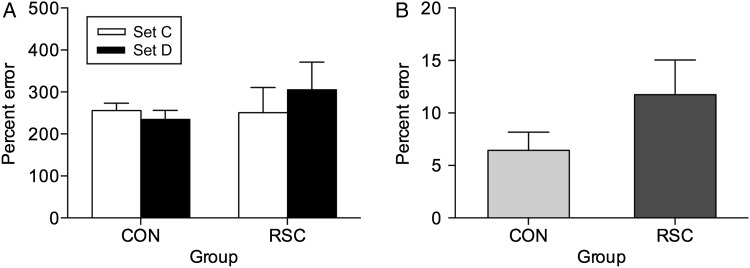
Postoperative new learning of new problems: “Across session learning.” (*A*) Total mean (+SEM) errors in learning 100 novel object-in-place scene discriminations to ≥90% correct across 2 consecutive testing sessions (Experiment 3) during the novel postoperatively learned set (Set D, black bars). The final preoperatively learned set (Set C, white bars) is included for comparison. (*B*) Total mean (+SEM) retention errors measured 24 h after achieving ≥90% learning criterion in the first testing session for the postoperatively learnt novel 100 object-in-place scene discriminations made by unoperated control monkeys (CON, *n* = 9) and bilateral retrosplenial cortex ablation monkeys (RSC, *n* = 4).

After relearning was completed, all monkeys learnt a new set (Set D) of 100 novel object-in-place scene discriminations presented in the same manner as the preoperatively acquired discriminations. Table 1 shows the sessions and errors made to reach criterion, set at ≥90% correct across 2 consecutive sessions. The one-way ANOVA with the between-subject factor “Group” (2 levels: RSC group, CON group) showed no significant difference in the number of errors made to reach criterion between the RSC and CON groups in this measure of anterograde amnesia, [*F*_1,11_ = 1.75, *P* = 0.213]. However, given that we had preoperative assessments of the individual monkeys′ learning abilities prior to neurosurgery, we also ran the one-way ANOVA with the between-subject factor “Group” (2 levels: RSC group, CON group) on the difference in score between errors-to-criterion in learning the last preoperative set acquired prior to surgery (Set C), and errors-to-criterion accrued during learning the novel postoperative set (Set D), which similarly showed no significant difference between the RSC and CON groups in this measure of anterograde amnesia, [*F*_1,11_ = 1.42, *P* = 0.259; see Fig. 7*A*]. Therefore, these analyses provide no evidence in support of the hypothesis that monkeys with bilateral RSC lesions are impaired at new learning of information learnt after brain injury.

The lack of a statistically significant new learning impairment in the analysis above may be deemed somewhat surprising given that deficits in learning new spatial memory processing paradigms are observed after RSC lesions in rodents (Vann and Aggleton 2002). Previously with macaques, Jones and Mishkin (1972) compared learning across a range of different performance criteria, and so for a more detailed analysis of new learning we adopted a similar approach here. As in the previous analysis, our measure assessed the difference in score between errors accrued in learning the last preoperative set acquired prior to surgery (Set C) and errors accrued during learning the novel postoperative set (Set D) but in this case only considering errors made before monkeys attained 65% correct performance across all problems reflecting above chance performance level. The one-way ANOVA with the between-subject factor “Group” (RSC vs. CON) showed only a nonsignificant trend [*F*_1,11_ = 3.87, *P* = 0.075]. Similar comparisons of learning rates for the difference in score to reach 70% correct, [*F*_1,11_ = 2.44, *P* = 0.146], 75% correct, [*F*_1,11_ = 3.90, *P* = 0.074], 80% correct, [*F*_1,11_ = 2.45, *P* = 0.146], or 85% correct, [*F*_1,11_ = 2.99, *P* = 0.112] were also all not significant. Table 3 shows the errors accrued by the RSC and CON group monkeys in learning to the 65, 70, 75, 80, and 85% correct together with the original 90% performance criteria. In summary, all these analyses taken together indicate no more than a numerical trend for RSC-lesioned monkeys to make more errors in new learning.

**Table 3:** New learning

Monkey	Set C						Set D					
	65%	70%	75%	80%	85%	90%	65%	70%	75%	80%	85%	90%
CON1	67	67	91	130	130	169	169	190	190	234	281	291
CON2	67	134	158	199	212	275	105	105	120	120	175	205
CON3	112	140	190	190	205	230	115	136	136	147	147	164
CON4	163	186	186	227	264	278	34	99	149	167	195	204
CON5	78	133	133	201	201	265	35	161	180	180	214	272
CON6	32	90	90	108	122	183	68	94	111	111	123	136
CON7	64	64	248	309	317	327	123	148	148	167	180	215
CON8	102	102	174	174	205	255	125	184	200	200	215	302
CON9	71	100	120	120	226	277	161	187	211	254	266	323
Mean	84	112.8	154.4	184.2	209.1	255.8	103.9	144.9	160.6	175.6	199.6	234.7
RSC1	75	103	155	220	242	410	161	185	185	205	246	289
RSC2	66	66	88	133	166	183	113	142	163	207	218	246
RSC3	32	56	56	98	111	138	105	105	105	151	151	191
RSC4	74	131	131	193	193	270	286	286	346	365	413	494
Mean	61.8	89	107.5	161	178	250.3	166.3	179.5	199.8	232	257	305

Note: Total errors made by individual monkeys during learning of a novel set 100 object-in-place scene discriminations.

The data shown are the total number of errors made to learn Set C (preoperatively learnt final set) and Set D (postoperative novel set) of 100 object-in-place scene discriminations to 65% correct, 70% correct, 75% correct, 80% correct, 85% correct in one session, and ≥90% correct across 2 consecutive sessions for unoperated control monkeys (CON1–CON9; CON1–CON5 are from a previously published study (Mitchell et al. 2008)) and monkeys with bilateral retrosplenial cortex ablations (RSC1–RSC4).

#### One-Trial Postoperative Retention Test of the 100 New Postoperatively Acquired Problems

Experiment 1 assessed postoperative retention of preoperatively acquired problems and revealed a deficit. In order to assess whether the deficit in retention generalizes to postoperative retention of postoperatively acquired problems, we analyzed retention errors made on the session after the monkeys attained ≥90% correct on the postoperative set of 100 problems (Set D). This comparison is essentially a one-trial retention test of the newly learned 100 object-in-place scene discriminations incorporating a 24-h delay. One-way ANOVA of errors made during this session for monkeys with RSC lesions and unoperated controls showed a significant main effect of Group, [*F*_1,12_ = 15.05, *P* = 0.003; see Figure 7*B*]. Monkeys with RSC lesions (mean = 11.75, SD = 3.30) made more errors than unoperated control monkeys (mean = 6.44, SD = 1.74) during the daily session that immediately followed (i.e., 24 h delay) their attaining ≥90% for the first time within a session during acquisition of Set D.

### Experiment 4: Anterograde Memory Assessment: Rapid Within-Session Postoperative New Learning of Sets of 10 New Object-in-Place Discriminations Each Day (One New RSC Animal Tested Pre- and Postoperatively)

**Figure 8.**
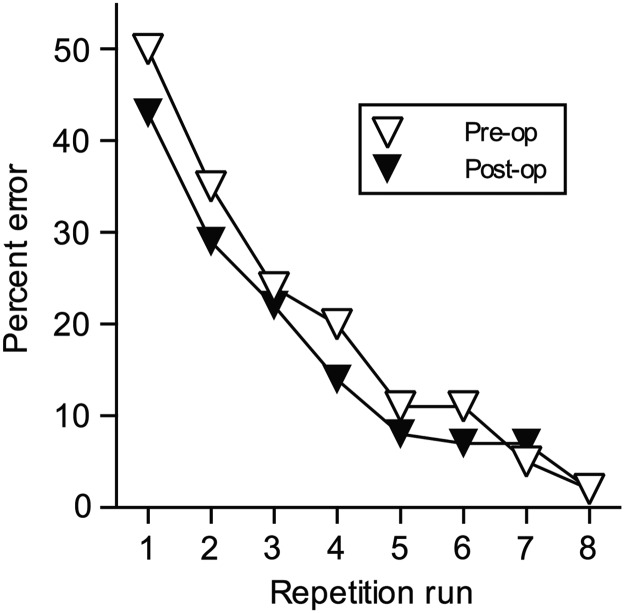
Postoperative new learning of new problems: “Rapid within” session learning. Mean percent error within-session learning curves (Experiment 4) for one monkey (RSC5) during learning of novel sets of 10 object-in-place scene discriminations with 8 repeats of 10 trials per session across the last 10 days of the within-session learning task, preoperatively (Pre-op), and postoperatively (Post-op) after bilateral retrosplenial cortex ablation (*n* = 1).

The aforementioned analyses all consider gradual learning across many days of large sets of object-in-place scene discriminations acquired through a combination of within-session and between-session learning. Previous studies have shown that components of the Delay–Brion (Papez) circuit impair rapid learning of small sets of object-in-place scene problems when novel scenes are used each day and which therefore also precludes between-session learning (Gaffan 1994; Parker and Gaffan 1997a, 1997b). Therefore, as a further preliminary assessment of anterograde amnesia after RSC lesions we assessed one monkey with a bilateral RSC lesion on a standard within-session object-in-place scene discrimination learning task. This monkey, RSC5 (Figs 2 and 3) showed no postoperative learning deficit for acquiring novel sets of 10 novel object-in-place scene discriminations with each new set learnt within a single daily session. We compared the monkey's preoperative versus postoperative within-session learning ability; Figure 8 plots the mean number of errors made per daily session on the 8 successive runs through the list of 10 novel problems that make up a session. Considering only the repetition runs (i.e., runs 2–8) averaged across the last 10 preoperative learning sessions, the mean errors per session made by RSC5 was 15.43 (SD = 11.59) compared with a mean of 14.71 (SD = 10.58) averaged across the 10 postoperative learning sessions. Figure 8 shows that monkey RSC5 performed numerically better postoperatively almost irrespective of run such that no analysis is required to determine whether there is a deficit. Given there was no hint of any impairment in this task in RSC5 we did not repeat this study in additional monkeys. Nonetheless, this experiment with a single monkey helps confirm that deficits in new learning of object-in-place scene discriminations are not a necessary consequence of RSC lesions.

## Discussion

Experiment 1 used one-trial postoperative retention tests for 300 preoperatively acquired object-in-place scene discrimination problems and showed that, as predicted, RSC damage in monkeys results in a retrograde memory deficit for preoperatively acquired object-in-place scene discriminations. The one-trial postoperative retention test procedure is designed to allow postoperative retention of preoperatively acquired memories to be assessed without any confound with relearning. Subsequently, in Experiment 2, we assessed relearning of the 300 problems by repeating the cycle of retention tests again 3 more times and the RSC group was unimpaired. Experiment 3 assessed whether RSC lesions had any effect on anterograde new learning of a set of 100 novel problems not seen preoperatively. Our data, across a range of performance criteria, indicated that new learning of these novel 100 object-in-place discriminations remained intact in the RSC group (a numerical trend for RSC monkeys to make more errors than control monkeys during learning remained nonsignificant in all learning-to-criteria analyses irrespective of performance criteria).

However, Experiment 3 also revealed, using a one-trial postoperative retention test, that monkeys with RSC lesions were significantly impaired at short-term retention (i.e., 24 h later) of the same postoperatively acquired set of 100 problems on the session after the set had been acquired to ≥90% performance criterion. This suggests that RSC lesions produce a general deficit in retention of well-learnt information, apparent in our experiments for both pre- and postoperatively acquired information. Further, Experiment 4 assessed whether the RSC lesion had any effect on anterograde new learning in the context of more rapid learning involving within-session learning exclusively; as the RSC lesion had no discernable effect we conclude that anterograde amnesia is not a necessary consequence of RSC lesions.

From our novel data collected for the first time in nonhuman primates, we conclude that a general mild deficit in retention of object-in-place memories underlies impaired performance in these learning and memory tasks in RSC-lesioned monkeys. We reason that this impaired performance results in a significantly greater deficit observed in retrograde amnesia than anterograde amnesia because intact new learning in RSC-lesioned monkeys can obscure mild retention loss during new learning.

Experiment 1 demonstrated that RSC lesions result in a robust retrograde memory deficit for preoperatively acquired object-in-place scene discriminations. The preoperatively acquired problems were divided into 3 sets of 100 problems with each set acquired successively. This design allowed us to assess whether the RSC lesion might produce a gradient of retrograde amnesia (i.e., better retention for more recently acquired versus remotely acquired problems) but the analyses only indicated a general retrograde retention deficit across all 3 sets. These retrograde memory deficits after RSC lesions in the monkey are consistent with similar nontemporally graded retrograde memory deficits after excitotoxic damage to RSC in rats tested in spatial maze and spontaneous object recognition paradigms (Haijima and Ichitani 2008, 2012). The finding also accords with previous studies that have also reported no evidence of a temporal gradient after rhinal or entorhinal lesions (Thornton et al. 1997; Mitchell et al. 2008), after fornix transection (Gaffan 1993a), or after combined damage to the magnocellular subdivision of the mediodorsal thalamus and fornix (Mitchell et al. 2008).

Experiment 1 incorporated one-trial postoperative retention tests to isolate retention deficits from any possible confound with relearning deficits whereas Experiment 2 examined subsequent performance across repeated cycles of retention testing thereby confounding retention and relearning but nonetheless allowing us to assess relearning in this content. We found that the RSC group was not significantly impaired at relearning, indeed the RSC group learnt at a rate numerically faster than controls (Fig. 6). Taken together, Experiments 1 and 2 suggest that RSC lesions might selectively impair retention and leave relearning intact. Experiments 3 and 4 sought to investigate this further by providing assessments of learning rates of novel information (i.e., new sets of object-in-place problems not seen preoperatively that were acquired either slowly within and across sessions in Experiment 3, or alternatively, acquired rapidly and solely within-sessions in Experiment 4). Experiments 3 and 4 both showed that new learning was intact in RSC-lesioned monkeys, although in Experiment 3 once monkeys had reached ≥90% correct criteria, the RSC-lesioned monkeys showed impaired retention of these discriminations when tested again on the same problems 24 h later. All the experiments taken together suggest that the underlying deficit in RSC-lesioned monkeys is one of retention of well-learnt information, not new learning of information per se.

The deficits we observed in Experiment 3 in short-term retention of postoperatively acquired object-in-place discriminations is consistent with similar observations of patients whose lesions include right RSC damage showing deficits in new visuo-spatial learning (Maguire 2001). Although the patients′ deficits typically resolved after several weeks (Maguire 2001), in patients there is the potential for reorganization of function to occur within their contralateral RSC. However, this possibility is not available in our bilaterally lesioned monkeys and we did not specifically assess if the deficits in our RSC-lesioned monkeys remained robust after several weeks. Although it must be noted that the retention deficit observed with the 24 h delay after monkeys had reach ≥90% criterion during postoperative new learning was at least 8 weeks post neurosurgery.

The current finding that RSC lesions in monkeys produce a deficit in retrograde amnesia greater than in anterograde new learning is consistent with a growing body of work indicating that for new learning (anterograde amnesia), damage to subcortical structures or interactions of cortical and subcortical structures appear to be particularly important in object-in-place scene discrimination learning paradigms (Gaffan 1993b, 1994; Gaffan et al. 2001, 2002; Mitchell et al. 2007, 2008; Buckley et al. 2008; Mitchell and Gaffan 2008; Browning et al. 2015). In contrast, previous studies have reported that damage limited to cortical sites significantly impairs retention, as assessed by one-trial postoperative retention tests; examples include rhinal (i.e., combined perirhinal and entorhinal) cortex lesions (Thornton et al. 1997), lesions limited to perirhinal cortex alone (Hampton and Murray 2002), lesions limited to entorhinal cortex (Mitchell et al. 2008) and lesions limited to TE within inferotemporal cortex (Dean and Weiskrantz 1974). Nonetheless, some cortical regions, for example, the entorhinal cortex (Charles, Browning et al. 2004a) and perirhinal cortex (Buckley and Gaffan 1998) also contribute substantially to anterograde amnesia.

Experiment 4 showed a lack of within-session object-in-place discrimination learning that is consistent with other monkey studies that have reported a lack of deficits in this task after aspiration lesions to the cingulate cortex that encompassed the anterior to posterior extent of the cingulate cortex located more dorsally and anteriorly to RSC (Parker and Gaffan 1997a) or after dorsolateral prefrontal cortex ablations (Baxter et al. 2008). In contrast, rodents with RSC lesions are impaired at an object-in-place learning task (Vann and Aggleton 2002). One possible account of this difference relates to the nature of the task differences between species; the macaque object-in-place scene learning task may be more taxing of allocentric spatial memory (relationship of stimulus items to each other) whereas the rodent object-in-place task may be more demanding upon both allocentric spatial memory and egocentric spatial memory (spatial relation with self) if the animal must also remember its own changes in location while performing this spatial task (for further discussion on the proposed importance of RSC for integrating different strategies see below).

Our study used aspiration lesions to damage the RSC, which would have caused damage to axons coursing through this region itself and may have caused inadvertent damage to fiber pathways in the cingulum bundle underlying cingulate cortex. Our histological examination revealed that one of our monkeys, RSC4, sustained unilateral damage to the cingulum bundle as a consequence of its aspiration lesion and this monkey made more errors during our postoperative performance measures than the other 3 monkeys (RSC1, RSC2, and RSC3) that did not have this unilateral cingulum bundle damage (Tables 1 and 3). These white matter fibers are certainly likely to be important for learning as they link distributed networks of structures involved in spatial cognition; this issue has been well considered previously in rodent studies with lesions in this region (Vann and Aggleton 2002). Indeed, several previous monkey studies from our laboratory also provide causal evidence to support the disconnection hypothesis as an explanation for amnesia and other cognitive deficits (Gaffan et al. 2001; Buckley et al. 2004, 2008; Charles, Gaffan et al. 2004b; Gaffan 2005; Mitchell et al. 2007, 2008; Browning et al. 2015).

These proposals are supported by evidence from the patient literature as well. Typically, in amnesic patients, the severity of retrograde memory impairments and anterograde memory impairments are poorly correlated (Kopelman 2000). Most clinical cases present with more pronounced anterograde amnesia than retrograde amnesia. However, in some cases (with damage to the medial temporal lobes or frontal lobes), retrograde amnesia can be more marked than anterograde amnesia (Bright et al. 2006; Hornberger et al. 2010; Smith et al. 2013). It is notable that some retrosplenial amnesics have both retrograde and anterograde amnesia. However, RSC damage in human cases is not precise and usually involves white matter tracts of the fornix and cingulum bundle and/or subcortical structures (Valenstein et al. 1987; Rudge and Warrington 1991). While anterograde amnesia after RSC atrophy in humans is possibly caused by this additional damage to these white matter pathways (disconnection accounts of amnesia) and/or subcortical structural damage, our study suggests that one cannot yet discount a contribution to anterograde amnesia from RSC cortical damage per se. Certainly, the present evidence shows that the RSC needs to be included, along with structures in the medial temporal lobes and frontal lobes, as one of a number of critical interconnected brain structure involved in learning and retention.

It may be argued that our preoperative retention test just prior to neurosurgery engaged some reconsolidation of the memories to confound our measure of retention. Our study is not able to discount this possibility. However, all monkeys showed differing levels of retention across the 3 sets during the preoperative retention test. During all of our retention tests all monkeys made more errors on Set A that was learnt more remotely in time (at least 2–3 months prior to surgery) than Set B (learnt at least one month prior to surgery) or Set C (learnt at least 1 week prior to surgery). Furthermore, across the 2 retention tests, even control monkeys showed levels of forgetting for the information that they had learnt most recently. Thus, whereas reconsolidation cannot be ruled out, it is certainly not the case that reconsolidation leaves all memory strengths equated.

The RSC is anatomically linked within an extended network of spatial memory related regions, and its key contribution to memory retention is likely generated from interactions between a range of prefrontal, parietal, and limbic cortical and interconnected subcortical structures as well. One notable proposal (Burgess et al. 2001; Vann et al. 2009) is that RSC mediates translation between egocentric-based inputs (self-generated movements) from parietal cortex that converge with allocentric based inputs and head direction information from within the extended hippocampal–diencephalic system (Taube 2007). Indeed, recent fMRI evidence that RSC is activated by permanent landmarks within the environment, perhaps because of their relative importance in helping with successful navigation (Auger et al. 2012), also accords with the linking or registering together of different sources of information (e.g., via useful landmarks) to aid the laying down of integrated episodic memories and their subsequent retrieval. Furthermore, RSC is activated during spatial navigation and so RSC may complement the role of the hippocampus and parahippocampal gyrus by updating egocentric and head direction information as the key features in the environment of the observer change (Iaria et al. 2007; Epstein 2008). Although our object-in-place paradigm was not designed to assess landmark-based encoding or retrieval, some elements of each scene are more prominent that others and may act as landmarks of potential use for separating otherwise relatively similar scenes and/or aiding monkeys′ encoding of objects. The fact that Experiments 3 and 4 showed no deficit in new learning suggests on the face of it that our RSC monkeys may have intact landmark-based encoding strategies and accordingly, a landmark-based retrieval deficit may indeed be consistent with our results. However, these suggestions remain speculative, as future work involving eye tracking would likely be required to assess whether monkeys postoperative new learning strategies were changed with respect to use of landmarks during encoding.

In humans, RSC is also activated in functional MR imaging studies when participants are asked to recall autobiographical memories or when they are asked to imagine the future (Buckner et al. 2008; Spreng et al. 2009). Our causal evidence indicates that the RSC in primates is important for retention even when an autobiographical memory component is not overtly apparent. Further, RSC lesions impair rodents’ abilities to shift spatial strategies (Vann et al. 2009). Deficits occur when rats switch from a reliance on extra-maze cues to intra-maze cues during memory performance (Vann et al. 2003; Vann and Aggleton 2004; Pothuizen et al. 2008) or when switching from a reliance on visual cues to nonvisual cues in light versus dark environments (Cooper and Mizumori 1999). The ability to integrate previous spatial information with current information may be significantly impeded following damage to the RSC. Similar types of information integration deficits are also apparent in rats after RSC damage in a response conflict paradigm, or spatial paradigms that require visual cues to be used flexibly across trials, or in cross-modal object recognition memory paradigms (Hindley et al. 2014a, 2014b; Nelson et al. 2014). It is also possible that our task may place some demands upon translation between egocentric and allocentric representations because the position from which the monkey views the scene in our task varies a little between trials according to the monkeys unrestrained sitting position, albeit within a limited range, in front of the fixed location of the scene displayed on the touchscreen. The development of RSC-mediated strategies to identify useful (i.e., relatively unique) landmarks may also be important during the course of laying down memories. Thus our current result accords with the notion that the RSC helps with registering or linking together related information from different spatial frameworks, modalities, and contexts, for the purpose of successful (episodic or otherwise) memory retrieval.

To conclude, our study, the first investigation of RSC lesions in monkeys, provides evidence that primate RSC makes a robust critical contribution to the retention of information. We also suggest that RSC lesions in primates may also contribute to the ability to integrate current task relevant information with previously acquired relevant information. It is clear from this evidence that a fuller understanding of how anterograde and retrograde memory loss relates to neural systems will require consideration of how a wider range of cortical and subcortical structures interact than previously and typically considered.

## Funding

This work was supported by a UK Medical Research Council Career Development Award (G0800329) to A.S.M. Funding to pay the Open Access publication charges for this article was provided by an RCUK Open Access Block Grant to University of Oxford.
